# Executive function and neural oscillations in adults with attention-deficit/hyperactivity disorder: a systematic review

**DOI:** 10.3389/fnins.2025.1617307

**Published:** 2025-07-15

**Authors:** Ziyao Su, Yingtan Wang, Bin Wang, Chuanliang Han, Haoran Zhang, Yanyan Gu, Yu Chen, Xixi Zhao, Yuwei Shi

**Affiliations:** ^1^The Second Affiliated Hospital of Xinjiang Medical University, Urumqi, China; ^2^Beijing Key Laboratory of Mental Disorders, National Clinical Research Center for Mental Disorders & National Center for Mental Disorders, Beijing Anding Hospital, Capital Medical University, Beijing, China; ^3^Advanced Innovation Center for Human Brain Protection, Capital Medical University, Beijing, China; ^4^School of Biomedical Sciences and Gerald Choa Neuroscience Institute, The Chinese University of Hong Kong, Shatin, China

**Keywords:** adult ADHD, electroencephalograph, resting-state, event related potential, neural oscillation

## Abstract

Attention-deficit/hyperactivity disorder (ADHD) is a childhood-onset neurobiological disorder that often persists into adulthood. Adult ADHD is an important public health concern due to its great social damage and challenges in clinical recognition, resulting in a significant disease burden. Nonetheless, the diagnosis of adult ADHD remains challenging due to the absence of specific symptoms and biological markers. The aims of this systematic review were as follows: (1) To discern whether there were any differences in resting-state electroencephalogram (EEG) and event related potential (ERP) between adult ADHD and healthy controls (HCs). (2) To ascertain whether ERP specific manifestations associated with executive function (EF) deficiencies. (3) To conduct an exploration into the mechanisms of specific electrophysiologic alterations. This review was conducted in PubMed-Medline and Web-of-Science from 1971 to August 15th, 2024 to summarize the EEG changes of adult ADHD. We focused on resting-state EEG to report spectral power across different frequency bands and ERPs under different experimental tasks, 68 studies were finally included. When studying the characteristics of resting-state EEG in adult ADHD patients, we observed that theta power exhibits a consistent upward trend. Congruous reduction Pe, P3, and N2 amplitudes during response inhibition tasks, with a further decrease in P3 and N2 amplitudes in sustained attention tasks. These EEG changes may stem from impairments in error detection, cognitive control, and attention allocation, meaning that core EFs are affected in adults with ADHD. Overall, consistent changes in resting-state EEG and ERPs could provide insight for the identification of ADHD in adults.

## 1 Introduction

Attention-deficit/hyperactivity disorder (ADHD) is a prevalent childhood-onset neurobiological disorder, nearly 60% of patients diagnosed in childhood continue to exhibit symptoms in adulthood (Anbarasan et al., 2020). The global prevalence of persistent adult ADHD was found to be 2.58%, while the prevalence of symptomatic adult ADHD was 6.76%. This equates to 139.84 million and 366.33 million affected adults respectively (Song et al., 2021). ADHD is associated with an increased risk of other psychiatric disorders, educational and occupational failure, accidents, criminal behavior, and additions (Amiri et al., 2020). Moreover, ADHD shows significant correlations with a wide range of comorbid psychiatric disorders, including depressive disorders, anxiety disorders, substance misuse, placing a considerable burden on patients, family and society (Austerman, 2015; Thapar and Cooper, 2016; Pagán et al., 2023).

The clinical presentation of ADHD tends to evolve and diminish across the developmental course (Adamou et al., 2020). Adult ADHD is relatively neglected in epidemiological studies compared with childhood ADHD (Sibley et al., 2016). Diagnosis can be challenging as symptoms are non-specific, researchers are attempting to find objective biological markers to help identify adult ADHD. Symptoms of adult ADHD include insufficient inhibitory control, defects in working memory, impaired socioemotional processing, and challenges in completing tasks that require sustained self-regulation of attention (Nijmeijer et al., 2008). Based on these observations, scholars proposed that executive function (EF) deficits which closely related to the frontal lobes may be the core features of ADHD (Nigg and Casey, 2005; Willcutt et al., 2005; Roth and Saykin, 2004; Barkley, 1997). EF is an umbrella term encompassing a range of higher cognitive processes that are both distinct and interrelated (Friedman and Miyake, 2017; Karr et al., 2018). There is currently no consensus on the classification of EFs. Some scholars believed four core EFs are conflict monitoring, response inhibition, set- shifting, and working memory updating (Miyake et al., 2000; Smit et al., 2023). There were still other researchers who divided EFs into cool and hot. According to them, cool-EFs impairment mainly manifests in response inhibition, working memory and cognitive flexibility, while hot-EFs impairment usually involves delayed gratification, reward/punishment-related decision-making, self-regulation, and emotion regulation (Willoughby et al., 2011).

Assessment of ADHD has been largely relayed on subjective reports from patients and clinical observations. Whether EEG could be utilized in clinical practice as a diagnostic aid to assist diagnosis or not, it would provide a potentially non-invasive and economical method with which to objectify the assessment process. There are a considerable number of studies for the use of electroencephalogram (EEG) in adult ADHD. In 2014 one published review (Lenartowicz and Loo, 2014) explored the use of EEG in diagnosing adult ADHD, concluding that EEG was not an appropriate diagnostic tool but has a potentially promising future. In 2020, based on the accession of 5 years of literature, it was concluded that EEG activities are potentially unique to adult ADHD, strongest support was derived for elevated theta and alpha activity (Adamou et al., 2020). In 2022, a systematic review suggested that resting-state and event-related modulation of alpha, beta and theta power, as well as the N2 and P3 components have potential for EEG measures, which can provide meaningful insights into the heterogeneity of ADHD (Slater et al., 2022).

Based on the background above, this systematic review focused on adult ADHD, innovatively categorized ERP studies according to the EFs domains, which adding conceptual clarity and enhanced clinical relevance. We included both resting-state EEG and event related potential (ERP) studies, providing a broad and integrated perspective on electrophysiological correlates of EF deficits. Objectives of this systematic review are as followed: (1) To discern whether there were any differences in resting state EEG and ERP between adult ADHD and healthy population. (2) To ascertain whether ERP specific manifestations associated with EF deficiencies. (3) To conduct an initial exploration into the mechanisms of specific electrophysiologic alterations.

## 2 Materials and methods

### 2.1 Search strategy and information sources

We conducted a comprehensive search of English-language literature from 1971 to August 15th, 2024, in publicly available datasets PubMed and Web of Science, there was no limitation on publication date in search strategy. The current review was performed in compliance with the Preferred Reported Items for Systematic Reviews and Meta Analyses (PRISMA) 2021 guidelines (Page et al., 2021) (PRISMA checklists see Supplementary Tables 6, 7). The following combinations of keywords were used in title and abstract: (”ADHD” OR “Attention Deficit Disorder with Hyperactivity”) AND (“EEG” OR “Electroencephalography” OR “Electroencephalogram” OR “Evoked Potential” OR “Event-Related Potential” OR “Wave”) AND (“Adult” OR “adult*” OR “Young adult”) AND (“Prospective studies” OR “Retrospective Studies” OR “Follow-up studies” OR “Cohort studies” OR prospective* OR retrospective* OR longitudinal* OR followup OR cohort*). In addition, hand searched of published systematic reviews, and references of the selected articles were undertaken.

### 2.2 Inclusion and exclusion criteria

The objectives and the inclusion criteria of this study were structured based on the elements of the PICOS model (Population of interest, Interventions, Comparators, Outcomes, and Study design), The specific items of inclusion and exclusion criteria have been listed in Table 1.

**TABLE 1:** Inclusion and exclusion criteria of studies.

Inclusion criteria	Exclusion criteria
Studies included at least one group of participants in adult ADHD	a. Studies included ADHD participants under 18 years old b. Studies included participants in comorbid with organic brain diseases (such as epilepsy, traumatic brain injury, neurodegenerative diseases etc.), or other serious physical illnesses c. Studies in lack of health control groups
Studies focused on resting state EEG or ERP	Studies employing PET, MRI or MEG
Studies concentrated on spectral power in different frequency bands of EEG and ERPs under different tasks	Studies exclusively focused on other EEG merits (such as asymmetry, coherence, event-related desynchronization, and event-related synchronization etc.)
Empirical studies	Reviews, cases, commentaries, or meta-analyses
Written in English	Written in other languages

### 2.3 Data extraction

Data extractions were conducted in duplicate by two independent reviewers, and discrepancies were resolved through discussion and consultation with a third reviewer. We roughly classify ERPs into five categories according to EFs, which are: Response Inhibition, Working Memory, Self-regulation of affects, Sustained Attention and others. From each included article was extracted and entered into tables, the extracted data included the following information: (1) authors and year of publication, (2) demographic characteristics (sample size, sex, age), (3) recording condition (eyes closed or eyes open), measures of frequency bands and range, spectral power type utilized (for resting state EEG), (4) Experimental task (for ERPs), (5) main findings. The selected articles and their data have been shown in the data extraction (Tables 2–9). In addition, other information such as the country, IQ, Co-morbidities condition and medication and rules of reduction/interruption are recorded in Supplementary Tables 1, 2, whereas the study outcomes were discussed in the results section. The analysis of the results has been generally explained in the discussion part.

**TABLE 2:** Main finding of the resting-state EEG studies included in the review.

Study	Delta	Theta	Alpha	Beta	Gamma
Kiiski et al. (2020a)	1–4 Hz ↑ Centro-parietal	4–8 Hz ↑ Centro-parietal	8–10 Hz ↑ Centro-parietal	Beta 1 13–16 Hz ↑ Frontal Beta 2 16–20 Hz ↑ Parietal	NA
Dupuy et al. (2021)	1.5–3.5 Hz NS	Relative 3.5–7.5 Hz ↑ Globally (male)	7.5–12.5 Hz NS	Absolute 12.5–25 Hz ↓ Globally (male)	35–45 Hz NS
Clarke et al. (2019)	1.5–3.5 Hz ↓ Frontal	3.5–7.5 Hz ↑ Globally	7.5–12.5 Hz NS	12.5–25 Hz NS	NA
Han et al. (2022)	NA	NA	Low alpha 8.42 ± 0.94 Hz NS Medium alpha 10.15 ± 0.76 Hz NS High alpha 11.81 ± 0.84 Hz NS	NA	NA
Tombor et al. (2019)	NA	NA	NA	NA	Absolute gamma 1 30.25–39 Hz ↓ gamma 2 39.25–48 Hz ↓ centroparietal
Li et al. (2019)	1–4 Hz NS	Relative 4–8 Hz ↑ globally	Relative 8–13 Hz ↓ globally	Relative 13–30 Hz ↑ central	NA
Schneidt et al. (2020)	NA	4–8 Hz NS	NA	13–21 Hz NS	NA
Bresnahan and Barry (2002)	2–4 Hz Absolute ↑ relative NS	4–8 Hz Absolute ↑ relative ↑	8–13 Hz Absolute ↑ relative NS	13–30 Hz Absolute ↑ relative NS	NA
Koehler et al. (2009)	1.5–3.5 Hz NS	A bsolute 3.5–7.5 Hz ↑	Absolute 7.5–12.5 Hz ↑	12.5–25 Hz NS	NA
Liechti et al. (2013)	NA	3.5–7.5 Hz NS	NA	12.5–25 Hz NS	NA
Markovska-Simoska and Pop-Jordanova (2017)	2–4 Hz NS	4–8 Hz NS	8–13 Hz NS	13–21 Hz NS	NA
Poil et al. (2014)	1–4 Hz NS	4–8 Hz NS	Absolute alpha 1 8–10 Hz ↑ alpha 2 10–13 Hz NS	Absolute 13–30 Hz ↑	30–45 Hz NS
Woltering et al. (2012)	NA	Absolute& relative 4–8 Hz ↑	Absolute& relative 8–12 Hz ↓	Absolute& relative 13–25 Hz ↓	NA
Buyck and Wiersema (2014)	NA	3.5–7.5 Hz NS	NA	Relative 12.5–25 Hz ↓ (Inattentive type)	NA
Clarke et al. (2008)	1.5–3.5 Hz absolute ↓	3.5–7.5 Hz relative ↑	7.5–12.5 Hz NS	Absolute 12.5–25 Hz Midline ↓ right posterior region ↑	NA
Kitsune et al. (2015)	0.5–3.5 Hz ↑ Time-1	3.5–7.5 Hz↑ Time-1	7.5–12 Hz NS	12–30 Hz ↑ Time-2	NA
Skirrow et al. (2015)	0.5–3.5 Hz NS	Relative 4–7.5 Hz ↑ frontal	7.5–12.5 Hz NS	12.5–30 Hz NS	NA
Yoon et al. (2024)	Absolute 1–4 Hz ↑ middle frontal	4–8 Hz NS	8–12 Hz NS	Absolute 12–30 Hz ↓ middle frontal	30–40 Hz NS
Loo et al. (2009)	NA	4–7 Hz NS	Absolute 8–12 Hz ↓	Absolute 12–20 Hz ↑	NA

↑, Frequency band power of ADHD higher than HC; ↓, Frequency band power of ADHD lower than HC; NS, Not significant change; NA, Not assessed. Absolute, absolute power of frequency band; Relative, relative power of frequency band; Time-1, Resting state before a 1.5-h cognitive task; Time-2, Resting state after a 1.5-h cognitive task.

**TABLE 3:** Event-related potential characterization of response inhibition.

Study	Task	Main finding
O’Connell et al. (2009)	Go/no-go task	Pe ↓
Czobor et al. (2017)	Go/no-go task	Pe ↓ ERN↓
Wiersema et al. (2009)	Go/no-go task	Pe ↓ ERN -
Rodriguez and Baylis (2007)	Go/no-go task	P3 ↓ (frontal site)
Kropotov et al. (2019)	Go/no-go task	P3 ↓
Woltering et al. (2013)	Go/no-go task	P3 ↓ N2 ↓
Münger et al. (2021)	Go/no-go task	Go P3 ↓ N2 ↓
Wiersema et al. (2006)	Go/no-go task	P3 ↓ N2 -
Münger et al. (2022)	Go/no-go task	Cue P3 ↓ N2d ↓ P3d ↓CNV ↓
Papp et al. (2020)	Go/no-go task	P1 ↓ (at occipital and inferotemporal areas)
Mayer et al. (2016)	Go/no-go task	CNV ↓
Karch et al. (2012)	Go/no-go task	Gamma ↑ (frontal and fronto-central area)
Herrmann et al. (2010)	Eriksen flanker task	Pe ↓ ERN↓ (In younger subsample, not elderly subsample)
Marquardt et al. (2018)	Flanker task	Pe ↓ ERN ↓ P3 ↓
McLoughlin et al. (2009)	Arrow flanker task	Pe - N2 ↓ Ne ↓
Ehlis et al. (2018)	Flanker task	ERN ↓ P300 ↓
Dubreuil-Vall et al. (2020)	Flanker task	Alpha (7–12 Hz) ↓ delta-theta (3–7 Hz) ↑
Smit et al. (2023)	Stroop task Stop-signal task	Theta -

Pe, error positivity; ERN, error-related negativity; CNV, contingent negative variation.

**TABLE 4:** Event-related potential characterization of sustained attention.

Study	Task	Main finding
Salomone et al. (2020)	Oddball task	P3 ↓
Marzinzik et al. (2012)	Visual oddball task	P3 ↓
Raz and Dan (2015b)	4-stimulus Oddball task	P3 ↓ N3 ↓
Barry et al. (2009)	inter-modal oddball task	P3 - N2 ↓ P2 ↑
Itagaki et al. (2011)	Auditory oddball task	P300 ↓
Micoulaud-Franchi et al. (2019)	Oddball task	P300 ↓
Leroy et al. (2018)	Oddball task	P350 ↓ N140 ↑
Raz and Dan (2015a)	Visual-emotional oddball task	P3 ↓ P1 ↑
Dhar et al. (2010)	O-X CPT (encompasses Go/no-go task)	P3 ↓
Münger et al. (2021)	Visual CPT (a classical Go/no-go task)	P3 ↓ N2 ↓
Kaur et al. (2019)	Visual CPT (a classical Go/no-go task)	P3 ↓ N2 ↓ N1 ↓
Münger et al. (2022)	CPT	CueP3 ↓ N2d ↓ P3d ↓ CNV ↓
Doehnert et al. (2013)	CPT	CNV ↓
Freichel et al. (2024)	CPT	Alpha -
Freichel et al. (2024)	CPT	Alpha -
Bozhilova et al. (2022)	SAT	Theta ↑ Alpha ↓ (occipital) Beta ↓
Skirrow et al. (2015)	CPT&SAT	Theta ↓
Cowley et al. (2022)	TOVA	P3 ↓ N2 ↓ theta ↓

CPT, Continuous Performance Task; TOVA, Test of Variables of Attention; SAT: Sustained attention task.

**TABLE 5:** Event-related potential characterization of working memory.

Study	Task	Main finding
Gu et al. (2018)	Visuospatial change detection task	CDA-
Spronk et al. (2013)	WM task	CDA ↓
Luo et al. (2019)	Visuospatial WM task	CDA↓ N2pc↓
Wiegand et al. (2016)	Visual short memory test	CDA ↓P3b ↑
Freichel et al. (2024)	Spatial delayed response WM task	Alpha -
Jang et al. (2020)	Spatial 2 back task	Theta↓ alpha↑
Smit et al. (2023)	n-back task	Theta –

WM: working memory; CDA: Contralateral Delay Activity.

**TABLE 6:** Event-related potential characterization of Self- regulation of affect.

Study	Task	Main finding
Shushakova et al. (2018a)	Visual image task and emotion regulation task	LPP ↑
Köchel et al. (2012)	Emotional version of a Go/no go task	LPP ↓
Balogh et al. (2017)	Emotional Go/no go task	ERN ↓ Pe ↓
Ibáñez et al. (2011)	Dual valence task	N170 ↓
Thoma et al. (2020)	Configural processing of emotional bodies and faces	N170 ↑ P250↑ P100↑
Shushakova et al. (2018b)	Verbal dot-probe task	P1 ↓ N2pc-
Raz and Dan (2015a)	Visual-emotional oddball task	P1 ↑ P3↓

LPP, Late positive potential.

**TABLE 7:** Event-related potential characterization of other Executive Function.

Study	Task	Main finding
Buyck and Wiersema (2015)	2-CRT task	Theta↑ beta↑
Cheung et al. (2017)	4-CRT task	P3↓
Herrmann et al. (2009)	Passive viewed pictures task (motivational-reward system)	EPN ↓
Mauriello et al. (2022)	Face- matching task	P200↓ N250↓P100- N170-
Gumenyuk et al. (2023)	Forced choice visual task (distraction)	RON ↓
Schneidt et al. (2018)	Two experimental tasks (distraction)	EPN↑ LPP↑
Hasler et al. (2016)	Attention Network Test	P3↓ CNV↓

CRT, Choice Reaction Time; RON/late negative: reorienting negativity; EPN, Early Posterior Negativity.

**TABLE 8:** Characteristics of the resting-state EEG studies included in the review.

Study	Subject	Sex	Age	Recording
Kiiski et al. (2020a)	38 ADHD 45 relatives 51 HC	19F 19M 10F 35M 29F 22M	27.1 ± 10.4 38.0 ± 14.2 29.9 ± 11.6	EC and EO
Dupuy et al. (2021)	32 ADHD 32 HC	16F 16M Each group	F 28.51 ± 1.38 M25.35 ± 2.03 F24.38 ± 2.67 M22.63 ± 2.22	EC
Clarke et al. (2019)	25 ADHD 25 HC	All male	21.69 ± 1.9 21.00 ± 2.2	EC
Han et al. (2022)	162 ADHD (141 persisters 21 remitters) 87 HC	88F 53M 3F 18M 29F 58M	25.41 ± 6.0 18.61 ± 0.87 23.96 ± 4.27	EC
Tombor et al. (2019)	42 ADHD (25MPH- 17MPH +) 59 HC	5F 20M 4F 13M 15F 44M	32.28 ± 10.35 28.94 ± 11.37 30.88 ± 11.00	EO
Li et al. (2019)	40 ADHD (IQ > 120) 40HC	17F 23M 14F 26M	25.85 ± 5.21 25.88 ± 3.83	EC
Schneidt et al. (2020)	113 ADHD 46 Subthreshold 42 HC	49F 64M 24F 22M 22F 20M	37.94 ± 11.08 38.41 ± 11.70 37.14 ± 11.50	EC and EO
Bresnahan and Barry (2002)	50 ADHD 50 non-ADHD 50 HC	25 F 25 M Each group	31.5 ± 9.2 34.0 ± 11.0 31.8 ± 8.9	EO
Koehler et al. (2009)	34 ADHD-C 34 HC	17F 17M Each group	33.26 ± 9.28 32.38 ± 8.99	EC
Liechti et al. (2013)	22 ADHD 21 HC	11F 11M 10F 11M	42.7 ± 4.4 44.0 ± 4.7	EC and EO
Markovska-Simoska and Pop-Jordanova (2017)	30 ADHD 30 HC	All male	**35.8 ± 8.65 35.3 ± 8.53**	EO
Poil et al. (2014)	22 ADHD 27 HC	12F 10M 17F 10M	37.9 ± 11.3 34.1 ± 10.5	EC
Woltering et al. (2012)	18ADHD 17HC	10F 8M 7F 10M	25.8 ± 4.27 24.4 ± 4.39	EC and EO
Buyck and Wiersema (2014)	26ADHD (15 ADHD-C 11 ADHD-I) 25HC	14F 12M 11F 14M	33.76 ± 10.17 35.32 ± 11.12	EC
Clarke et al. (2008)	20 ADHD (13 ADHD-C 7 ADHD-I) 20HC	All male	21.7 20.3	EC
Kitsune et al. (2015)	76 ADHD 85 HC	8 F 68 M 1F 84M	18.70 ± 2.91 18.29 ± 1.76	EC and EO
Skirrow et al. (2015)	41 ADHD 48 HC	All male	28.5 ± 9.5 29.0 ± 10.4	–
Yoon et al. (2024)	51 ADHD 52 HC	3F 48M 8F 44M	21.16 ± 2.56 20.94 ± 2.22	EC
Loo et al. (2009)	38 ADHD 42 HC	20F 18M 21F 21M	45 ± 6.0 46 ± 5.4	EC &EO&CPT

F, Female; M, Male; HC, Health Control; EC, Eyes Close; EO, Eyes Open; ADHD-C, ADHD combined type; ADHD-I, ADHD inattentive type; ADHD-H, ADHD hyperactive/impulsive type; MPH, Methylphenidate.

**TABLE 9:** Characteristics of the event-related studies included in the review.

Study	Subject	Sex	Age
Cowley et al. (2022)	53 ADHD 18 HC	28F 25M 12F 6M	36.26 ± 10.22 32.78 ± 10.82
Herrmann et al. (2010)	34ADHD-C (youngster subgroup elderly subgroup) 34HC (youngster subgroup elderly subgroup)	8F 9M 8F 9M 7F 10M 7F 10M	25.2 ± 4.4 40.9 ± 6.8 24.2 ± 3.1 39.7 ± 6.6
Marquardt et al. (2018)	27 ADHD 28 HC	11F 16M 16F 12M	35.32 ± 8.8 33.37 ± 7.0
Dubreuil-Vall et al. (2020)	20 ADHD 20 HC	10F 10M 10F 10M	43.85 ± 14.78 29.90 ± 10.77
McLoughlin et al. (2009)	21 ADHD (17 ADHD-C 4 ADHD-I) 20 HC	All male	32.51 ± 5.84 30.00 ± 6.51
Buyck and Wiersema (2015)	24 ADHD (15 ADHD-C 9 ADHD-I) 20 HC	11F 13M 9F 11M	34.38 ± 10.21 36.55 ± 11.21
Ehlis et al. (2018)	34 ADHD 34 HC	13F 21M 18 F 16M	30.29 ± 9.47 27.62 ± 7.43
Papp et al. (2020)	26 ADHD (16 ADHD-C 7 ADHD-I 3 ADHD-H) 25 HC	8F 18M 6F 19M	28.9 ± 8.4 27.3 ± 5.0
Kropotov et al. (2019)	63 ADHD (42 ADHD-C 18 ADHD-I 3 ADHD-H) 132 HC	33F 20M 79F 53M	33.1 ± 7.84 31.8 ± 8.26
Rodriguez and Baylis (2007)	16 ADHD-C 16 ADHD-I 16 ADHD-H 16 HC	34F 30M	19.5 ± 1.94
Bozhilova et al. (2022)	23 ADHD 25 HC	10F 13M 13F 12M	36.73 ± 8.67 31.80 ± 11.42
Münger et al. (2022)	210 ADHD 158 HC	103F 107M 108F 50M	35.1 ± 10.1 32.5 ± 12.0
Wiersema et al. (2006)	19 ADHD 19 HC	All male	32.1 ± 12.3 31.2 ± 11.0
Woltering et al. (2013)	65 ADHD 32 HC	33F 32M 18F 14M	25 ± 5.8 25 ± 4.9
Münger et al. (2021)	447 ADHD 227 HC	151F 296M 133F 94M	16.8 ± 13.7 20.6 ± 14.1
Mayer et al. (2016)	23 ADHD (5 ADHD-C 18 ADHD-I) 22 HC	9F 14M -	36.57 ± 12.67 36.41 ± 12.14
Wiersema et al. (2009)	23 ADHD 19 HC	10F 13M 8F 11M	29.3 ± 11.0 30.9 ± 11.0
Balogh et al. (2017)	26 ADHD (7 ADHD-C 12 ADHD-I 7 ADHD-H) 14 HC	6F 20M 3F 11M	26.7 ± 5.7 31.5 ± 11.4
Czobor et al. (2017)	22 ADHD-C 29 HC	5F 17M 10F 19M	39.6 ± 9.7 30.1 ± 9.0
Köchel et al. (2012)	15 ADHD 15 HC	All male	26.67 ± 3.44 25.73 ± 3.24
O’Connell et al. (2009)	18 ADHD-C 21 HC	2F 16M 1F 20M	23.7 ± 5.1 22.0 ± 2.9
Karch et al. (2012)	24 ADHD 30 HC	10F 14M 14F 17M	33.6 ± 10.00 34.3 ± 10.98
Smit et al. (2023)	27 ADHD (7 ADHD-C 20 ADHD-I) 22 no diagnosis 21 HC	21F 6M 14F 8M 14F 7M	30.0 ± 7.3 35.0 ± 10.5 32.0 ± 12.1
Luo et al. (2019)	32 ADHD-I 34 HC	14F 15M 9F 21M	26.51 ± 5.41 25.05 ± 2.79
Jang et al. (2020)	40 ADHD 41 HC	33F 7M 30F 11M	21.35 ± 1.87 21.58 ± 2.13
Ibáñez et al. (2011)	10 ADHD (8 ADHD-C 2 ADHD-I) 10 HC	9F 1M 9F 1M	33.1 ± 3.6 33.0 ± 3.8
Thoma et al. (2020)	18 ADHD 25 HC	11F 7M 14F 11M	37.1 ± 10.2 35.0 ± 11.0
Herrmann et al. (2009)	32 ADHD 32 HC	15F 17M 15F 17M	33.0 ± 9.9 31.9 ± 9.6
Shushakova et al. (2018a)	39 ADHD (21 ADHD-C 18 ADHD-I) 40 HC	18F 21M 18F 22M	31.21 ± 8.27 31.08 ± 8.83
Shushakova et al. (2018b)	39 ADHD (22 ADHD-C 17 ADHD-I) 41 HC	18F 21M 17F 24M	31.15 ± 8.24 30.59 ± 8.95
Salomone et al. (2020)	51 ADHD 28 HC	11F 40M 9F 19M	32.78 ± 10.96 30.6 ± 10.3
Marzinzik et al. (2012)	15 ADHD 15 HC	10F 5M 9F 6M	29.9 ± 7.7 32.4 ± 7.3
Raz and Dan (2015a)	17 ADHD 20 HC	14F 3M 14F 6M	24.07 ± 1.73 24.52 ± 2.87
Raz and Dan (2015b)	21 ADHD 19 HC	16F 5M 15F 4M	25.42 ± 2.11 24.72 ± 2.72
Barry et al. (2009)	18 ADHD 18 HC	All male	21.9 ± 1.8 20.6 ± 2.1
Itagaki et al. (2011)	54 ADHD 40 HC	37F 17M 19F 21M	31.9 ± 6.5 31.1 ± 6.7
Leroy et al. (2018)	14 ADHD (5 ADHD-C 14 ADHD-I) 14 HC	4F 10M 4F 10M	38 ± 13 32 ± 9
Kaur et al. (2019)	35 ADHD 35 HC	7F 28M 6F 29M	20.3 ± 1.12 20.6 ± 1.28
Dhar et al. (2010)	16 ADHD 16 HC	All male	33.1 ± 8.5 33.7 ± 8.9
Freichel et al. (2024)	85 ADHD 105 HC	18F 67M 28F 77M	44.31 ± 6.14 44.07 ± 6.02
Doehnert et al. (2013)	11ADHD 12 HC	1F 10M 4F 8M	21.91 ± 1.46 21.12 ± 1.29
Cheung et al. (2017)	93 ADHD-C 174 HC	-	18.28 ± 2.98 17.76 ± 2.16
Mauriello et al. (2022)	23 ADHD 23 HC	10F 13M 10F 13M	24.2 ± 3.7 23.3 ± 3.8
Gumenyuk et al. (2023)	9 ADHD 9 HC	6F 3M 6F 3M	22.3 ± 4.42 22.3 ± 4.48
Schneidt et al. (2018)	36 ADHD 37 HC	17F 19M 20F 17M	36.81 ± 10.82 37.00 ± 11.43
Hasler et al. (2016)	21 ADHD 20 HC	7F 14M 13F 7M	40.05 ± 9.5 25.5 ± 4
Wiegand et al. (2016)	16 ADHD (8 ADHD-C 8 ADHD-I) 16 HC	9F 7M 10F 6M	30.0 ± 9.8 30.4 ± 9.8
Spronk et al. (2013)	17 ADHD (8 ADHD-C 9 ADHD-I) 16 HC	8F 9M 8F 8M	31 ± 8.8 28.2 ± 5.9

### 2.4 Study quality assessment

Two raters independently assessed study quality using the modified Newcastle Ottawa Scale (NOS). The detailed criteria of modified NOS in Supplementary Table 9. The case–control studies subscale was used for assessing the risk of bias. NOS provides three domains: (1) selection, (2) comparability and (3) exposure. The highest score is 9. A score from 9 to 7 indicates high quality, from 6 to 4 moderate quality, and from 3 to 0 low quality.

## 3 Results

### 3.1 Literature search and assessment of risk of bias

**FIGURE 1:**
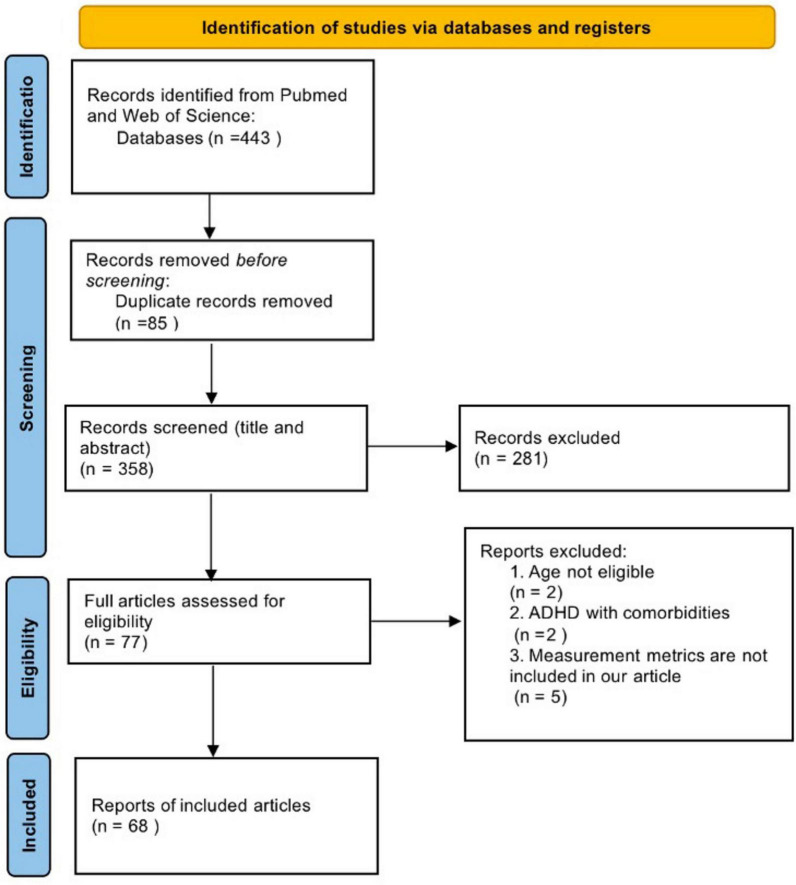
Preferred reporting items for systematic reviews and meta-analyses (PRISMA) flow diagram of study selection.

A total of 443 articles were initially identified from Pub-Med and Web-of-Science databases using our search terms (Figure 1), 85 duplicate articles were removed. After reading titles and abstracts, 281 articles were excluded. Upon further reading the full text, 9 articles were excluded, including 2 studies that ADHD participants under 18 years old. Adult ADHD in two articles have comorbidities. Five studies did not utilize the measurements we were intended to include. Ultimately, 68 articles were included, and the mean score of quality assessment of the 68 studies was 5.9, indicating a moderate quality (quality assessment of included studies see Supplementary Table 8).

### 3.2 General character of the study

#### 3.2.1 Resting state spectral power

**FIGURE 2:**
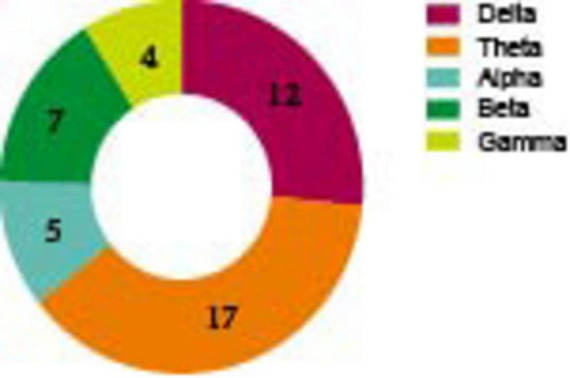
The number of different frequency bands among included studies (resting state).

**FIGURE 3:**
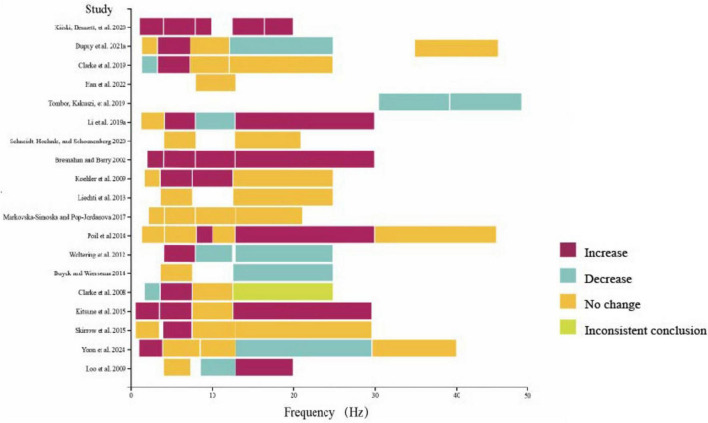
The changes of different frequencies in included studies (resting state).

In the resting state (Table 2; Figure 2), the most investigated frequency band was theta (17 studies), 10 of them had consistently elevated results (the specific changes of frequencies during resting state see Figure 3). Three articles manifested that theta power increased across the whole brain (Dupuy et al., 2021; Clarke et al., 2019; Li et al., 2019). One included study indicated that theta power increased in Centro-parietal area (Kiiski et al., 2020b), another concluded that relative theta power increased in frontal region (Skirrow et al., 2015).

The least studied frequency band was gamma, with only 4 studies involved, among which 3 showed no significant difference between adult ADHD and HC, while one study suggested a decrease in gamma band power in right centroparietal region (Tombor et al., 2019).

The measurement results of delta, alpha, and beta frequency bands are not consistent. For delta frequency band of adult ADHD, 4 included studies indicated increases delta power compared to HCs, whereas 2 articles indicated decreased delta activities (Clarke et al., 2008; Clarke et al., 2019). Significantly, Bresnahan and Barry in 2002 indicated that absolute delta power of adult ADHD patients was higher compared to HC, but there was no significant difference in relative delta power (Bresnahan and Barry, 2002).

For alpha band of adult ADHD, 4 studies showed an increase in band power, 3 studies suggested that adult ADHD has lower alpha power than HCs. 8 studies showed no significant difference in alpha power between ADHD and HC in adults. Kiiski and Bennett in 2020 drew conclusions that theta power of adult ADHD was increased in centro-parietal region (Kiiski et al., 2020a). Li in 2019 indicated that the relative alpha power was decreased across the whole brain (Li et al., 2019).

The results of researches in beta waves are most heterogeneous among all frequencies, 7 articles showed an increase in band power, while 5 studies indicated decreased beta activities. Interestingly, one study found that beta power (13–16 Hz) increased in frontal region, and beta activities (16–20 Hz) became higher in parietal region in adult ADHD (Kiiski et al., 2020a). Another study indicated that absolute beta power decreased in midline but increased in right posterior region (Clarke et al., 2008). Yoon in 2024 reached the same outcome: adult ADHD have lower absolute beta power in midline to HC (Yoon et al., 2024).

It is worth noticing that the classification of five main frequency bands have subtle differences, and some articles further divided them into sub-bands (Fig. 3 indicated the frequency bands ranges and changes of each study). Among them, 3 studies indicated sub-component of specific frequency band in patients with ADHD were consistent (Kiiski et al., 2020a; Han et al., 2022; Tombor et al., 2019). One study founded alpha 1 (8–10 Hz) and alpha 2 (10–13 Hz) have inconsistent changes of adult ADHD participants compared to HCs (Poil et al., 2014).

#### 3.2.2 Main experimental tasks and ERPs of EFs

**FIGURE 4:**
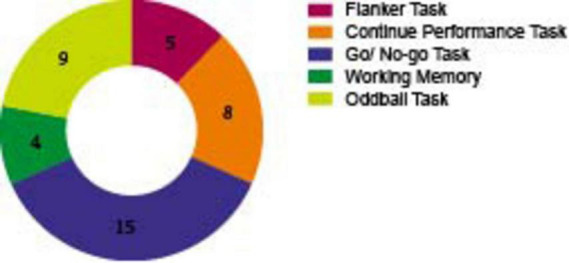
Number of included studies by experimental tasks. The chart summarizes the experimental tasks used during EEG recording in the included studies. (Tasks that are performed less than 3 times are not included).

**FIGURE 5:**
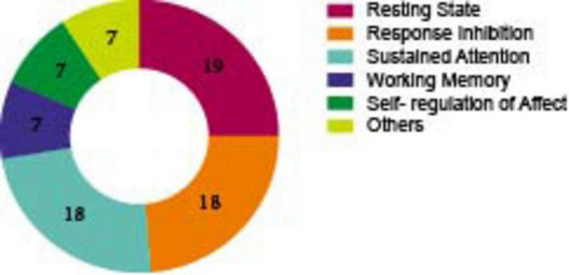
Number of included studies by EFs.

Figures 4, 5 and Supplementary Tables 3, 5 separately show the main experimental paradigms and EFs of included studies (The exact numbers are marked in Figures 4, 5).

As demonstrated in Figure 4, a variety of experimental paradigms were employed in the assessment of EFs. Of these, the Go/No-Go paradigm was conducted in 15 studies, thus rendering it the most frequently used. In this paradigm, participants are required to respond to frequent “GO” stimuli but withhold responses to rare “NO-GO” stimuli, it has been used to assess response inhibition, sustained attention, and self-regulation of affect. The Oddball task was the second most used, with 9 studies adopting it. In this task, participants need to detect rare target stimuli (“oddballs”) embedded in a sequence of frequent standard stimuli. It particularly involves selective attentional processes, which are defined as the ability to focus on goal-relevant events while ignoring irrelevant information. The Continuous Performance Test (CPT) was utilized in 8 studies. This well-established behavioral task is designed to investigate response inhibition and sustained attention. Participants are required to respond to specific target stimuli while inhibiting responses to non-targets over a prolonged period. The Flanker task was implemented in 5 studies. In this test, participants are required to identify a central target stimulus while ignoring flanking distractors that may be congruent or incongruent with the target, it is another task that involves inhibition function.

In addition, working memory tasks were adopted in 4 studies. In these tasks, participants temporarily store and manipulate visual (e.g., shapes, colors) and spatial (e.g., locations, movements) information (The describe and related EFs of paradigms of ERPs see Supplementary Table 3).

As illustrated in Figure 5, 18 of these researches evaluated response inhibition in adult ADHD patients, among them, 12 articles employed the Go/No-Go experimental paradigm, 5 articles utilized the Flanker task, and 1 study adopted the Stroop and Stop-Signal task. Of the 18 articles that evaluated the sustained attention in participants with ADHD, the oddball task and the CPT task were the most frequently used (8 each). Seven studies reported on the assessment of working memory, two of which utilized the N-back experimental paradigm. Seven studies discussed the self-regulation of affect, two employed the emotional go/no go experimental paradigm. Since some experiments cannot be categorized among the four executive functions that we have divided, we classify them as “other execution function (Table 7).

**FIGURE 6:**
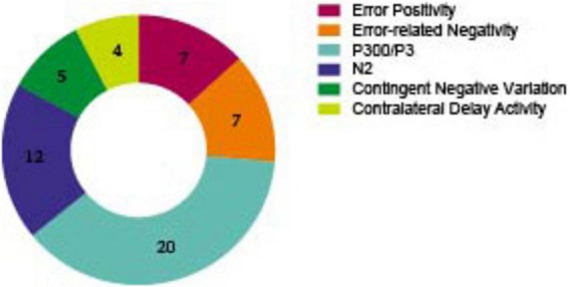
Number of included studies by EEG measurements. (measurements that are recorded less than 3 times are not included).

Figure 6 and Supplementary Table 4 illustrates the ERPs involved in our included studies (the exact numbers are marked in Figure 6), of which the most used is P3 (P300, P3d, P3b, GoP3 etc.) in 20 studies. Among them, six are for response inhibition, 12 are for sustained attention, one is for working memory and one is for self- regulation of affect. The second most used ERP metric is N2 (N2pc, N2d) in 12 studies, of these, five each on response inhibition and sustained attention, one each on working memory and self- regulation of affect. After that, Error Positivity (Pe) was recorded in seven studies, of which six were employed for the purpose of detecting response inhibition and one for the assessment of self-regulation of affect. It is important to note that all Contralateral Delay Activity (CDA) presents in the assessment of WM. Measurements that are recorded less than 3 times were not included.

## 4 Discussion

This systematic review sought to explore EEG alterations in ADHD adults across both resting and event-related states. We are the first to categorize the ERP results in ADHD patients based on their EF performance across diverse tasks. Our goal is to identify neuro-electrophysiological markers associated with specific dimensions of EF deficits and to explore potential neuropathological mechanisms behind these changes. The findings indicate that ADHD adults tend to demonstrate a consistent increase in theta band power compared to HCs when in a resting state. Additionally, during response inhibition tasks, the amplitudes of ERP components such as Pe, P3, and N2 were consistently lower in ADHD patients. During sustained attention tasks, there were more pronounced reductions of P3 and N2 amplitudes in ADHD patients than in controls.

### 4.1 Increased theta oscillation in the resting state

The outcomes of literatures included in this review indicate that ADHD adults exhibit heightened theta band power relative to HCs. Although the specific mechanism remains unclear, two hypotheses—the maturational lag hypothesis and the cortical hypo-arousal hypothesis—may provide insight into this manifestation (Ji et al., 2022; Byeon et al., 2020). According to the maturational lag hypothesis, slow-wave activity typically diminishes with age, whereas fast-wave activity increases and is eventually predominant. In children with ADHD, however, a delay in brain development has been observed, characterized by increased slow-wave activity (delta and theta waves) and decreased fast-wave activity (alpha and beta waves) (Clarke et al., 2019; Mann et al., 1992). Previous studies have found that approximately 65% of ADHD children continue to exhibit symptoms in adulthood (Lara et al., 2009). It is crucial to determine whether children with ADHD maintain delayed brain development into adulthood or if these delays represent a persistent dysfunction that extends from childhood to adulthood. An 11-year longitudinal study conducted by Clarke found that children ADHD patients exhibited increased relative theta wave power and decreased alpha wave power at the whole brain level compared to normal children. In adulthood, elevated theta waves persist in ADHD patients compared to controls, although the degree of EEG abnormalities is less pronounced than in childhood. These findings suggest that adult ADHD patients still experience a lag in brain development, supporting the maturational lag hypothesis (Clarke et al., 2019).

Some scholars have interpreted the increased slow-wave activities like delta and theta bands as indicative of a cortical low arousal state (Satterfield and Cantwell, 1974; Rowe et al., 2005; Satterfield and Dawson, 1971). The neurochemical mechanisms underlying ADHD are thought to involve a complex set of imbalances in different neurotransmitters and neural networks, in particular the “overactivity” in inhibitory interneurons within the neocortex and the thalamic reticular nucleus (TRN), which may contribute to low levels arousal. The increase in interneuron activity associated with slow-wave activity can be further explained by the activation of cholinergic and/or norepinephrine (NA) metabolic receptors with inhibitory effects (Rowe et al., 2005).

Clinical studies have shown that impulsivity and inattention symptoms are still prevalent in adults with ADHD, although hyperactivity symptoms tend to diminish with age (Biederman et al., 2000). According to a study investigated EEG changes of ADHD in patients of different age groups (children, adolescents, and adults), researchers found that beta activity decreased while theta activity increased with age, aligning with clinical observations. Based on these findings, Bresnahan et al. proposed that beta activity may be associated with hyperactivity, whereas theta activity may be linked to impulsivity (Bresnahan et al., 1999). The low arousal model suggests that the increased theta power in adults with ADHD reflects a low arousal state of the central nervous system. ADHD compensatory symptoms, such as susceptibility to external distractions, difficulty in concentrating, and manifestation of hyperactivity, can be a way of trying to stimulate the nervous system.

In the included articles, 10 out of 17 studies reported an increase in theta band power, while 7 researches indicated no significant change in this frequency band of adult ADHD patients when compared to HCs. We noticed that the participants’ age in unchanged studies are higher than elevated theta studies. For the ages of participants in the included literature, ADHD patients was greater than 18 years, and there was no upper limit. Herrmann and his colleges divided the adult ADHD into two subgroup, youngster subgroup and elderly subgroup, the mean age of youngster group is 25 and for elderly is 40. EEG outcomes changes in youngster group but not in elderly samples (Herrmann et al., 2010). This result may indicate some ADHD related deficits vanish with age. Furthermore, according to the maturational lag hypothesis and the cortical hypo-arousal hypothesis, ADHD patients’ brain undergoes compensatory changes that may result in symptom relief and theta waves normalized.

In addition, the classification of ADHD exerts certain influences on EEG results. This disease typically categorized into inattentive, predominantly hyperactive/impulsive, and combined subtypes based on clinical manifestations. In a systematic review published recently, researcher indicated that resting state and task-related modulation of EEG and ERPs are different among ADHD subtypes (Slater et al., 2022). Buyck and Wiersema indicated fast wave activity decrease in inattentive subtype, but not in Combined subtype (Buyck and Wiersema, 2015).

Furthermore, gender, intelligence quotient (IQ), and recording context may further influence the results of studies. Several studies have found that male adults with ADHD are more likely to exhibit elevated theta band power (Clarke et al., 2008; Skirrow et al., 2015). Comparisons between high-IQ (IQ ≥ 120) adult ADHD patients and HCs have also shown that theta relative power is elevated globally (Li et al., 2019). In 2015, Kitsune et al. tried to investigate the impact of recording context difference on the results, recording the resting state before (Time-1) and after (Time-2) a 1.5-h cognitive task, they found that theta power only increased at Time-1 (Kitsune et al., 2015).

### 4.2 Decreased Pe, P3, and N2 in response inhibition

In the included studies of this systematic review, adult ADHD patients consistently exhibited a decrease in the amplitude of Pe, P3, and N2 components compared to HCs during tasks related to inhibition function. This suggests that adult ADHD patients may have deficiencies in inhibitory control. Pe typically reaches peak at centro-parietal sites around 200–450 ms after the occurrence of the erroneous response, which is thought to be an ERP that associated with erroneous responses, signifying conscious recognition of error (Falkenstein et al., 1991). The abnormal decrease in Pe may be due to reduced activation of the anterior cingulate cortex (ACC) (O’Connell et al., 2009). ACC is a critical area for effective error handling (Ridderinkhof et al., 2004), which plays a key role in complex cognitive processes (object detection, response selection, error supervision, and reward-based decision-making) (Bush et al., 2002). Numerous functional magnetic resonance imaging (fMRI) studies have found that ACC dysfunction exist in ADHD patients (Rubia et al., 1999; Schulz et al., 2004). Additionally, studies have shown that functional connectivity between ACC and other brain regions reduced in ADHD patients (Castellanos et al., 2008).

The P3 typically peaks at 300–600 ms after stimulation (Cycowicz and Friedman, 2004; Debener et al., 2005), and the component is thought to be associated with attention resources allocation for task implementation (Marquardt et al., 2018). Previous studies have shown that adult ADHD patients not only exhibit a decrease in P3 amplitude but also demonstrate a higher rate of response errors and increased reaction time variability during inhibition-related tasks. The amplitude of the P3 component is negatively correlated with clinical symptom levels (Marquardt et al., 2018). Furthermore, higher IQ levels are associated with fewer missed errors, shorter reaction times, and larger No-Go P3 amplitudes (Münger et al., 2021), suggesting that high IQ ADHD patients may have more attention resources. The decrease in P3 amplitude observed in ADHD patients during inhibition-related tasks may indicate that fewer attentional resources are allocated to inhibitory control and related assessment processes (Woltering et al., 2013).

N2 is a negative potential located in the frontal-central region, typically measured at 200–400 ms, and is thought to be associated with conflict monitoring, response inhibition and selection (Wiersema and Roeyers, 2009). The neural sources of anterior N2 are primarily believed to originate from ACC, an area closely associated with conflict monitoring and attention control (Bekker et al., 2005). Previous studies have demonstrated that N2 amplitude in adult ADHD patients correlates with ADHD symptoms (reduced amplitudes associated with more serious symptoms) (Woltering et al., 2013). This suggests that lower N2 amplitude may be linked to poorer inhibitory control and self-regulation in ADHD patients. These findings indicate that reduced N2 amplitude in adult ADHD may reflect underlying deficits in inhibitory control and self-regulation, which are critical for managing attention and behaviors (Wiersema and Roeyers, 2009).

### 4.3 Decreased P3, N2 in sustained attention

Adult ADHD patients consistently show reductions in the amplitude of P3 and N2 components compared to HCs when completing sustained attention related tasks. The decreased amplitude of P3 component may reflect deficits in attention, stimulus processing, and evaluation abilities, or an inappropriate attentional resources allocation (Barry et al., 2009; Itagaki et al., 2011; Jonkman et al., 2004). These alterations may be due to impaired connectivity between cognitive control network and default mode network in ADHD (Castellanos et al., 2008). However, Barry et al. have not observed changes in P3 amplitude among adult ADHD patients during the oddball task, they proposed that this might result from patients’ high concentration during tasks, which may partially compensate for ADHD information processing deficits (Barry et al., 2009). N2 components, particularly posterior scalp N2, were thought to be involved in conflict monitoring (Münger et al., 2022). In situations requiring multitasking or suppression of distracting information, a decrease in N2 amplitude may indicate that patients are less effective at resolving conflicts or inhibiting distractions. According to literatures included in this systematic review, adult ADHD patients consistently show reductions in N2 amplitude when completing sustained attention tasks (Barry et al., 2009; Münger et al., 2021; Kaur et al., 2019), suggesting a potential disorder in conflict monitoring. This impairment may make it difficult for adults with ADHD to sustain attention over prolonged periods.

### 4.4 Inconsistent results

However, current studies on the resting-state alpha and beta oscillations in adult ADHD have not reached a consistent conclusion. Some investigators have found that alpha power is higher in adult ADHD patients compared to HCs (Bresnahan and Barry, 2002; Poil et al., 2014; Kiiski et al., 2020b; Koehler et al., 2009). According to these researchers, increases or decreases in alpha power reflect states of cortical inhibition or excitability, respectively (Haegens et al., 2011). Elevated alpha power may be associated with a decreased ability to process stimuli, leading to inhibition of attention or increased distractibility (Mathewson et al., 2009). Conversely, other researchers have found that alpha-band power is reduced in adult ADHD patients compared to controls (Woltering et al., 2012; Loo et al., 2009; Li et al., 2019), these researchers suggest that decrease in alpha power may indicate a reduction in cortical inhibition, which can lead to increased subcortical signaling. This may conversely bring about behavioral manifestations such as excitability, impulsivity, and hyperactivity (Sauseng et al., 2013).

In addition, some studies have found that beta band power in adults with ADHD is higher than that in HCs. Researchers hypothesize that this increase in beta band activity may be associated with heightened cerebral cortex activity and impaired emotion control (Clarke et al., 2001, 2011). Specifically, in children with ADHD, beta hyperactivity is more likely to associated with mood fluctuation and aggressive behaviors. Furthermore, Li and colleagues observed an increase in the relative power of beta frequency band in adult ADHD patients at rest and noted that this change may be strongly associated with emotion regulation disorders, particularly emotional instability and rapidly changing emotional states (Li et al., 2019). Jaworska et al. recorded resting state EEG data from 14 adult patients with ADHD, 14 anger control disorder and 14 HCs, the results indicated that the ADHD patients exhibited higher beta wave activities. Researchers believe this may reflect a chronic state of hypervigilance associated with the development of anger emotions (Jaworska et al., 2013). However, some investigators have reported decreased beta power in adult ADHD patients compared to HCs (Dupuy et al., 2021; Yoon et al., 2024). This finding is consistent with the trend of decreasing ADHD symptoms with age, suggesting that beta activity may be associated with the severity of ADHD symptoms (Bresnahan et al., 1999). In summary, the results regarding beta power changes are not entirely consistent, which may be related to various clinical subtypes of ADHD and evolution of clinical symptoms with age growing.

### 4.5 Limitation

Based on a comprehensive analysis of the included literatures, we observed that adult patients with ADHD demonstrated consistent EEG characteristics and changes in ERP components in the context of specific EFs compared to HCs. However, there were significant inconsistencies results across studies. These differences may be attributed to various confounding factors, Tables 8, 9 and Supplementary Tables 1, 2 summarize the general characteristics, experimental paradigms of studies included in this review.

Firstly, in term of general demographic characters, several studies have reported divergent EEG changes due to differences in gender, age, IQ. and subtype. Other than that, ADHD patients have high frequency of co-morbidities, this condition is unavoidable. Some studies have recorded combination with other psychiatric disorders (see Supplementary Tables 1, 2), but the nomenclature and reference criteria are divergent thus cannot be unified. Moreover, methylphenidate (MPH) as a central nervous system stimulant is commonly prescribed as a first-line medication for ADHD. Tombor and KKuaszi reported MPH treatment was associated with increased gamma activity compared to MPH naïve (Tombor et al., 2019). Bresnahan found there was reduction in slow wave activity in ADHD patients receiving stimulant medication compared to untreated group (Bresnahan et al., 2006). A meta-analysis demonstrated MPH tend to increase P300 amplitude in individuals with ADHD, contributing to normalize brain activity (Barroso et al., 2025). Medication use for ADHD and durations of medication refrainment were recorded in Supplementary Tables 1, 2. The findings demonstrated inconsistency results to whether medication influenced EEG outcomes. Therefore, future researchers should focus on standardizing diagnosis and screened criteria and controlling for confounding factors, and conducting long-term longitudinal studies to track disease progression.

Secondly, the subgroup of particular component of ERPs and its correlation with cognitive domains has been inconsistent across studies (see Supplementary Table 4). Thirdly, in terms of experimental design, many experimental paradigms involve multiple EFs assessment (see Supplementary Table 3 and Supplementary Figures 1, 2), it is difficult to define exactly which tasks are specifically related to working memory, response inhibition, sustained attention, or self-regulation of affect. It is recommended that future endeavors focus on refining the meanings and roles of ERPs components, with the object of correspondence with explicit cognitive processes.

## 5 Conclusion and future direction

In summary, resting-state EEG of adult ADHD patients is characterized by an increase in theta band power, which may be consistent with the cortical low arousal model. During functional tasks, especially the response inhibition tasks, ADHD patients tend to exhibit a significant reduction in the amplitude of ERP components such as Pe, N2, and P3. These reductions may reflect deficiencies in error detection and control functions. Additionally, reduction in P3 and N2 components during sustained attention tasks suggests that ADHD patients may have difficulties in effectively allocating attentional resources. However, these findings should be interpreted with caution due to the presence of numerous confounding factors in the studies. Of note, this systematic review did not perform a meta- analysis, primarily because of heterogeneous protocols, inconsistent reporting and clinical diversity. To advance the field, future research should standardize methodologies, comprehensive clinical documentation and control confounders. Through these efforts, the field can establish robust electrophysiological biomarkers for ADHD, ultimately improving diagnostic precision and effective interventions.

## Data Availability

The original contributions presented in the study are included in the article/Supplementary material, further inquiries can be directed to the corresponding authors.
