# Supplementary material for: Executive function and neural oscillations in adults with attention-deficit/hyperactivity disorder: a systematic review
Source: Front Neurosci. 2025 Jul 15;19:1617307. doi: 10.3389/fnins.2025.1617307 (PMC12303959; doi:10.3389/fnins.2025.1617307)
Supplement: Supplementary file 1 [file Data_Sheet_1.pdf]

81Supplement table 1. Characters of the Resting-state EEG studies included in the review

Study& country	Diagnosis criteria& assessment method	IQ（ADHD，HC）	Co-morbidities	medications & rules of reduction/ interruption
(Kiiski, Bennett, et al. 2020), Ireland	Co-morbidities screened: Shortened version of the Structural Clinical Interview DSM-IV.<br>ADHD symptom severity assessment: CAARS.<br>IQ estimated: National Adult Reading Test.	113.1 ±10.5<br>114.0 ±6.8	1 depression, 3 anxiety, 1 mild Asperger's syndrome, 3 dyslexia, 1 dyscalculia.	14 MPH +, 3 lisdexamfetamine<br>Abstained from medication for at least 24h prior to EEG data collection.
(Dupuy et al. 2021a), Australia	Co-morbidities screened: DSM-V adult ADHD diagnostic criteria.<br>ADHD symptom severity assessment: CAARS.<br>IQ estimated: WAIS-III.	105.94 (F) 110.25 (M)<br>112.38 (F) 112.31 (M)		30 ADHD participants had been unmedicated for a minimum period of 5 years prior to this study.<br>Abstained from medication for at least 24h prior to testing.
(Clarke et al. 2019), Australia	Diagnosed and co-morbidities screened: DSM-V diagnostic criteria.<br>Assessment for depression: CES-D.<br>IQ estimated: WAIS-III and Woodcock Reading Mastery Test.	105.2 ±11.8<br>113.4 ±12.4		Abstained from medication for at least 24 h prior to testing.
(Han et al. 2022), China	ADHD diagnosis and symptom assessment: CAARS and SCID.	118.34±12.31<br>120.23 ±9.91		All ADHD participants were medication naïve.
(Tombor et al. 2019), Hungary	ADHD symptoms assessment: DSM-IV structured interview.<br>ADHD symptom severity assessment: CAARS.			25 MPH-,<br>17 MPH+
(Li et al. 2019a), China	ADHD diagnosis: CAADID based on DSM-IV.<br>Co-morbidities screened: SCID.	128.18± 5.25<br>126.35 ± 4.96	4 anxiety, 2 obsessive compulsive disorder, 1 bipolar, 1 social phobia, 1	All ADHD participants were medication naïve.

	IQ estimated: Full-Scale WAIS Revised in China.<br>EF Measurement: Behavior Rating Inventory of Executive Function		substance abuse, 6 history of depression	
(Schneidt, Hoehnle, and Schoenenberg 2020), Germany	ADHD diagnosis: German ASRS, DSM-IV Personality Disorders Questionnaire and German version of the Wender-Reimherr Interview.<br>ADHD symptom assessment.: CAARS, Barratt-Impulsiveness-Scale, State Trait Anxiety Inventory and State Trait Anxiety Inventory.<br>Comorbid disorders assessment: Beck Depression Inventory, Mini International Neuropsychiatric Interview.<br>IQ estimated: computerized versions of the Vocabulary Test and Wiener Matrizen Test.<br>EF measurement: Inventory for Complex Attention, Stroop Color-Word Test, and the Test for Attentional Performance – Flexibility.	107.66±9.85<br>109.50± 10.44	9 Affective disorders, 9 current MDE, 24 anxiety disorders, 2 eating disorder.	14 ADHD medication, 20 antidepressants, 9 Selective serotonin reuptake inhibitors, 5 Selective serotonin noradrenalin reuptake inhibitors, 3 Tricyclic antidepressants, 68 Psychotherapeutic treatments in the past.<br>Participants were no or stable use of medication for at least two months.
(Bresnahan and Barry 2002), Australia	ADHD diagnosis and assessment: DSM-IV criteria, Barkley's Semi-structured Interview, scores on the Wender Utah Rating Scale and the Symptom Checklist-90-R.<br>Comorbid disorders assessment: subjects were excluded if there was evidence of Axis 1 or 2 disorders or other comorbid disorders.			All subjects free from substance use and/or current psychoactive medication.
(Koehler et al. 2009), Germany	ADHD diagnosis and symptom assessment: ICD-10, DSM-IV, ASRS.			MPH+ (the number of participants was not mention）<br>Abstained from medication for at least 3 days prior to the test.

(Liechti et al. 2013), Switzerland	ADHD diagnosis: ASRS<br>IQ assessment: German WAIS subtests vocabulary and block design.	111.7± 11.9<br>112.9 ±12.9	Reading difficulties ADHD: 10; HC: 3.	All participants were free of psychotropic medication and patients on stimulant medication suspended treatment at least 48 h before testing.
(Markovska-Simoska and Pop-Jordanova 2017), Republic of Macedonia	ADHD diagnosis: Barkley's Semi-structured Interview			No one of the examined individuals was taking psychostimulants.
(Poil et al. 2014), Switzerland	ADHD symptoms assessed: CAARS.<br>IQ assessment: WIE-III.	107.9±17.8<br>111.6 ±16.5	1 Reading and writing problems, 1 Isolated Phobia	All did not take antidepressants drugs, 10 MPH +, 3 Ritalin, 2 Concerta, 1 Symbicord, 1 Cannabis.<br>Interrupt medication at least 48 h before testing.
(Woltering et al. 2012), Canada	ADHD symptoms assessed and comorbidities screened: CAARS, Adult ASRS<br>EF measurement: WAIS-IV		3 learning disability, 1 anxiety and depression.	6 stimulants, 2 stimulants and antidepressants, 2 stimulants, antidepressants, and other non-prescriptive medications.
(Buyck and Wiersema 2014), Belgium	ADHD diagnosis: DSM-IV criteria.<br>Symptom severity assessment: ADHD Rating Scale-IV.<br>IQ assessment: WAIS-III.	109.96 ±12.16<br>111.96 ±10.87		12 ADHD using stimulants were asked to refrain from medication 48 h before experiment.
(Clarke et al. 2008), Australia	ADHD diagnosis: DSM-IV criteria, clinical interview and AD/HD rating scales.<br>ADHD symptoms and co-comorbid assessment: CAARS, GHQ.<br>IQ assessment: WAIS-Reading Mastery Test	107.4<br>114.1.		All subjects were treated with Ritalin exclusively, and the length of treatment varying from between 1.5 years to 10 years (mean duration 5 years 3 months).

				Refrain from using ADHD related medication for 24 h prior to being tested.
(Kitsune et al. 2015), UK	ADHD diagnosis: DSM-IV criteria, Diagnostic Interview for ADHD in Adults.<br>IQ assessment: WAIS-IV vocabulary and block design subtests.	98.44±14.27<br>111.67 ±12.86		Refrain from using ADHD related medication for 48h before research session.
(Skirrow et al. 2015), UK	ADHD diagnosis: CAADID for DSM-IV, CAADID. Any axis I or II comorbid psychiatric diagnosis; history of axis I psychiatric disorders were excluded.<br>Symptoms assessment: Barkley Adult ADHD rating scale, and the Centre for Neurologic Study-lability scale.<br>IQ: WAIS.	108.3±13.8<br>112.3 ±13.4		21 ADHD MPH+ and took medication on the day of their follow-up assessment. Treatment was maintained for a minimum of 2.5 months. 1 dexamphetamine, 1 atomoxetine.
(Yoon et al. 2024), Korea	ADHD diagnosis: DSM-5 diagnostic criteria, ASRS.<br>EF assessment: According to Korean WAIS-IV.	90.88± 10.73<br>93.02 ± 9.91	24 depression disorder, 6 anxiety disorder.	2 antidepressant, 3 anxiolytics
(Loo et al. 2009), USA	Comorbidities screened: Schedule for Affective Disorders and Schizophrenia.<br>IQ assessment: WAIS- III	116 ±14.3<br>116 ±12.6	24 any mood diagnosis, 20 any anxiety diagnosis, 11 oppositional defiant, 16 substance abuse.	Subjects on stimulant medication were asked to discontinue use for 24 hours prior to their visit.

Supplement table 2. Characteristics of the event-related studies included in the review

Study& country	Procedure	IQ (ADHD, HC)	Co-morbidities	Medication
(Cowley, Juurmaa, and Palomäki 2022), Finland	ADHD diagnosis: ASRS, Adult ADHD Diagnostic Interview<br>IQ assessment: WAIS IV			Interrupt medication at least 48 h before testing.
(Herrmann et al. 2010), Germany	ADHD diagnosis: DSM-IV symptom list for ADHD, WURS			
(Marquardt et al. 2018), Norway	ADHD diagnosis: ASSR<br>IQ assessment: Wechsler-Abbreviated Scale of Intelligence	111.25 ± 11<br>115.18 ± 9	13 anxiety/depression, 1 autism spectrum disorders, 2 bipolar disorder, 2 alcohol-related problems, 2 drug-related , 8 other mental health problems, 4 eating disorders	14 Stimulants were asked to refrain from medication 48h prior to investigation. Other types of medication (antihistamines, melatonin antihypertensive, contraception, antidiabetics, asthma medication, statins, thyroxines) were taken as prescribed.
(Dubreuil-Vall, Ruffini, and Camprodon 2020), USA	ADHD diagnosis: according to DSM-5<br>Symptom severity assessment: Adult ASR		Psychosis, bipolar disorder, substance use disorder and neurological conditions were exclusion criteria.	Patients discontinued 2 days prior to the experiment
(McLoughlin et al. 2009), UK	ADHD diagnosis: Barkley Adult ADHD rating scale, criteria for DSM-IV<br>IQ assessment: WAIS-II	118±10<br>122±12	Exclusion criteria for the ADHD group included the presence of an Axis I or II co-morbid psychiatric diagnosis and taking any psychoactive medication other than stimulant medication for treatment of ADHD	Interrupt medication at least 48 h before testing.
(Ehlis, Deppermann, and Fallgatter 2018), Germany	ADHD diagnosis and symptom assessment: DSM-IV criteria, German ASRS, SCID, Beck Depression Inventory II<br>IQ assessment: Mehrfachwahl-Wortschatz Intelligenztest	115.7 ± 14.6<br>118.6 ± 15.7		6 methylphenidate, 1 dexamphetamine, 1 atomoxetine and 1 bupropion. ADHD medication was discontinued 2 to 7 days before the trial. All subjects in the group had been treated exclusively using Ritalin, with treatment duration varying between 1.5 years and 10 years
(Papp et al. 2020), Hungary	ADHD diagnosis: DSM-IV diagnostic criteria, CAARS<br>Co-morbidities screened: SCL-90R			9 MPH medication monotherapy, 3 bupropion (1 monotherapy, 1 antidepressant combination, 1 combined with MPH)

(Kropotov et al. 2019), Russia	ADHD diagnosis and assessment: DSM-IV criteria, Brief Symptom Inventory, Current and Childhood Symptoms Scales			Interrupt medication at least 24h before testing.
(Rodriguez and Baylis 2007), USA	ADHD diagnosis and assessment: DSM-IV checklist			9 Adderall or Ritalin<br>Interrupt ADHD treatment medication 12-24h prior to testing.
(Bozhilova et al. 2022), UK	ADHD diagnosis: met both DSM-IV and DSM-V ADHD criteria, Diagnostic Interview for ADHD<br>IQ assessment: WASI-II	111.50 ± 13.25<br>114.28 ± 16.72	7 anxiety and/or depression.	12 stimulants, 2 atomoxetine.<br>Interrupt medication at least 48h before testing.
(Munger et al. 2022), Switzerland	ADHD diagnosis: according to the DSM-5<br>IQ assessment: Wiener Matrizen-Test 2	99 ± 16<br>105 ± 14		
(Wiersema et al. 2006), Belgium	ADHD diagnosis: ADHD (DSM oriented) scale, ASR,<br>IQ assessment: WAIS-III	104<br>107	Approximately 21% of the adults with ADHD met criteria for comorbid antisocial personality disorder	ADHD patients stopped taking methylphenidate (9) or other stimulants (1) 48 h before participation. The use of anti-depressive medication (4) was not stopped.
(Woltering et al. 2013), Canada	ADHD diagnosis and symptom assessment: ASRS, cognitive failures questionnaire, Symptom Assessment-45			20 stimulants, 1 anti-depressants, 4 combination of stimulants and antidepressants.<br>participants were not asked to stop or change their medication treatment
(Münger et al. 2021), Switzerland.	ADHD diagnosis: according to the DSM-5<br>IQ assessment: standard IQ paper and pencil test	109±**13**<br>102±**15**		All subjects have been medication-free on the day of the assessment
(Mayer, Wyckoff, and Strehl 2016), Germany	ADHD diagnosis: DSM-IV, SCID<br>IQ assessment: Culture Fair Intelligence Test	109.73 ± 12.17<br>113.38± 9.73		Interrupt medication at least 24h before testing.
(Wiersema, van der Meere, and Roeyers 2009), Belgium	ADHD diagnosis: ASR<br>IQ assessment: WAIS-III	107±**15.2**<br>**105±13.5**		
(Balogh et al. 2017), Hungary	ADHD diagnosis: CAARS		2 depression, 1 dysthymia, 1 somatization disorder, 1 panic disorder	Interrupt medication at least 10h before testing.
(Czobor et al. 2017), Hungary	ADHD diagnosis: DSM-IV criteria<br>Comorbid disorders assessment:90-item Symptom Check List<br>ADHD symptom severity assessment: CAARS、ASRS			15 MPH, 1 antidepressant

(Köchel, Leutgeb, and Schienle 2012), Austra	ADHD diagnosis and symptom severity assessment： WURS, Wender–Reimherr-Interview, Trait Meta Mood Scale			
(O'Connell et al. 2009), Ireland	ADHD diagnosis： SCID of DSM-IV<br>ADHD symptom severity assessment: CAARS， WURS	108 ±10.5<br>113 ±11	1 life-time depression, 1 current depression, 1 bipolar disorder, 1 current anxiety disorder, 4 substance abuse	9 stimulant medication, 4 stimulant medication in the past but had stopped, 5 were stimulant-naive.<br>Patients were withdrawn from any stimulant medication 36h prior to testing.
(Karch et al. 2012), Germany	ADHD diagnosis: based on DSM-IV criteria for CAARS<br>IQ assessment: California Verbal Learning Test			
(Smit et al. 2023a), Netherlands	ADHD diagnosis and symptom assessment: based on the DSM-IV criteria,<br>Beck Depression Inventory II<br>EF assessment: Executive Function-Adult version		11 participants reported other types of diagnoses (e.g., autism spectrum disorder, eating disorder, mood disorder, post-traumatic stress disorder).	
(Luo et al. 2019) , China	ADHD diagnosis: SCID (DSM-IV)<br>IQ assessment: WAIS	120±7<br>122±5	1 social phobia, 1 had obsessive-compulsive disorder, 1 posttraumatic stress disorder, 2 dysthymia disorder	
(Jang, Kim, and Kim 2020), Korea	ADHD diagnosis: ASRS, CAARS, SCID (DSM-IV)<br>IQ assessment: Korea WAIS	111.23 ± 9.70<br>113.88 ± 9.08		
(Ibáñez et al. 2011), Argentina	ADHD diagnosis and symptom severity assessment: fulfilled DSM-IV criteria, Depression Inventory II<br>IQ assessment: WAIS III			All patients were taking MPH, which was suspended on the day of ERP recordings.
(Thoma et al. 2020), Germany	ADHD diagnosis: fulfilled diagnostic criteria (DSM-IV)<br>Symptom assessment: German version of the Beck Depression Inventory, Social Interaction Anxiety Scale, Social Phobia Scale, German adaptation of the Wender-Reimherr Interview		3 autism spectrum, 2 currently diagnosed alcohol use disorder, 1 bipolar disorder	9 MPH, 1 selective serotonine noradrenaline reuptake inhibitor. 1 tricyclic antidepressant
(Herrmann et al. 2009), Germany	ADHD diagnosis and symptom assessment: ICD-10、DSM-IV			Patients discontinued all medication at least 3 days prior to investigation

(Shushakova, Ohrmann, and Pedersen 2018), Germany	ADHD diagnosis: ccording to the DSM-5, ASSR,<br>Symptom severity assessment: Wender Utah Rating Scale-German Short Version, BDI-II Beck Depression Inventory-II<br>IQ assessment: WAIS-IV		2 mild dysthymic disorder, 1 mild social phobia, 1 eating disorder.	7 stimulant medication, 3 taking stimulants in combination with antidepressants (2 selective serotonin reuptake inhibitors; 1 selective serotonin-norepinephrine reuptake inhibitor)
(Salomone et al. 2020), Ireland	ADHD diagnosis: CAADID (DSM-IV), CAARS, WURS<br>IQ assessment: WAIS-III	111.23 ±6.01<br>113.09 ±5.03	7 history of depression, 5 current depression, 5 history of anxiety, 4 current anxiety disorder, 3 substance abuse	21 psychostimulant medication for ADHD<br>Interrupt medication at least 24h before testing.
(Marzinzik et al. 2012), Germany	ADHD diagnosis and symptom assessment: CAADID (DSM-IV), ADHD-Checklist			All ADHD participants were medication naïve.
(Raz and Dan 2015a), Israel	ADHD diagnosis: ARS, satisfaction of the diagnostic criteria of DSM-IV for adult ADHD			7 Ritalin, 1 Concerta
(Barry et al. 2009), Australia	ADHD diagnosis: CAARS, according to DSM<br>Symptom assessment: GHQ-60, CAARS, Center for Epidemiological Studies–Depression Scale<br>IQ assessment: WAIS	104.5 ± 9.4<br>110.0 ± 13.9		
(Itagaki et al. 2011), Japan	ADHD diagnosis: satisfied the diagnostic criteria of DSM-IV,			2 MPH+, 5 anti-depressant drugs, 4 antianxiety agents, 2 hypnotics
(Micoulaud-Franchi et al. 2019), France	ADHD diagnosis and symptom assessment: CAADID, ASRS, Trait Anxiety Inventory			8 MPH+
(Leroy et al. 2018), Belgium	ADHD diagnosis and symptom assessment: CAADID		1 depression, 1 anxiety, 1 dysthymia, 1 bipolar disorder	Interrupt medication at least 48h before testing.
(Kaur et al. 2019), India	ADHD diagnosis: according to DSM-5<br>Comorbid disorders assessment: Mini International Neuropsychiatric Interview, ASRS			All ADHD participants were medication naïve.
(Dhar et al. 2010), Belgium	ADHD diagnosis: according to DSM-IV, ASR<br>IQ assessment: abbreviated version of the Groninger Intelligentie Test	110.3 ± 9.6<br>116.4 ± 9.4		

(Doehnert et al. 2013) Switzerland	ADHD diagnosis: German adaptation of the Wender–Reimherr Adult Attention Deficit Disorder Scale, ASR based on DSM-IV,			Interrupt medication at least 48h before testing.
(Cheung et al. 2017), UK	ADHD diagnosis: DSM-IV, Barkley's functional impairment scale<br>IQ assessment: WASI			Interrupt medication at least 48h before testing.
(Mauriello et al. 2022), Switzerland	ADHD diagnosis and symptom severity assessment: ASRS, State–Trait Anxiety Inventory			16 MPH,<br>Interrupt medication at least 24h before testing.
(Gumenyuk et al. 2023), USA	ADHD diagnosis: adult ASRS			5 Adderall, 2 Wellbutrin, 2 Strattera, 1 Guanfacine, 1 Vyvanse, 1 MPH, 1 Lamictal.<br>Interrupt medication at least 12h before testing.
(Schneidt et al. 2018), Germany	ADHD diagnosis and symptom severity assessment: CAARS, Barratt Impulsiveness Scale, Beck Depression Inventory-II, Homburger ADHD Scales for Adults		11 depression, 9 anxiety disorders and 2 eating disorders	3 MPH, 3 antidepressants (selective serotonin reuptake inhibitors, serotonin norepinephrine reuptake inhibitors).
(Hasler et al. 2016), Switzerland	ADHD diagnosis: according to DSM-IV-TR criteria, ASRS, Wender Utah rating scale			Interrupt medication at least 48h before testing.
(Wiegand et al. 2016), Germany	ADHD diagnosis: according to DSM-IV、CAARS<br>Symptom Severity assessment: Beck Depression Inventory<br>IQ assessment: Wender Utah Rating Scale.	98.1 ± 12.9<br>102.7± 10.4	5 mild and 2 moderate depression levels. 3 further diagnosed as suffering from dyslexia	2 selective serotonin re-uptake inhibitors and were not required to interrupt medication. 5 were taking methylphenidate, but were off<br>medication for at least 24 h prior to participating in the experiment.
(Spronk, Vogel, and Jonkman 2013), USA	ADHD diagnosis: ASR, DSM-IV<br>IQ assessment: block design and vocabulary tests of the WISC and WAIS.	103± 10<br>97.9± 9.3		

DSM：Diagnostic and Statistical Manual of Mental Disorders；CAARS: The Conners' Adult ADHD Rating Scale；IQ：Intelligence Quotient；CAADID：Conners Adult ADHD Diagnostic Interview for DSM；MPH: methylphenidate; WAIS: Weschler Adult Intelligence Scale; CES-D: Centre for Epidemiologic Studies Depression Scale; GHQ: General Health Questionnaire; SCID: Structured Clinical Interview for DSM; EF: executive function; ICD: International Statistical Classification of Diseases and Related Health Problems; ASRS: Adult ADHD Self-Report Scale; WURS: Wender Utah Rating Scale;

Supplement table 3. Main paradigms of excluded studies

Paradigm	description	Related ERPs	Related EF
Flanker Task	Participants are required to identify a central target stimulus while ignoring flanking distractors that may be congruent or incongruent with the target.	Pe, ERN, P3, N2, Ne	Response Inhibition
Continue Performance Task	Participants must respond to specific target stimuli while inhibiting responses to non-targets over a prolonged period.	P3, N2, N1, CNV	Sustained Attention<br>Response Inhibition
Go/ No-go Task	Participants must quickly respond to frequent "Go" stimuli，but withhold responses to rare "No-Go" stimuli.	Pe, ERN, P3, N2, CNV, LPP	Response Inhibition<br>Sustained Attention<br>Self- regulation of Affect
Working Memory Task	Participants temporarily stores and manipulates visual (e.g., shapes, colors) and spatial (e.g., locations, movements) information.	CDA, N2, P3	Working Memory
Oddball Task	Participants detect rare target stimuli ("oddballs") embedded in a sequence of frequent standard stimuli.	P3, N3, N2, P2, P350, N140, P1	Sustained Attention<br>Self-regulation of Affect

Supplement table 4. Main ERPs of included studies

ERP	Definition	description	Related task	Related EF
P3/P300	Positive deflection occurring approximately 300ms after stimulus onset	Relates to recruitment and resource allocation necessary for task performance	Flanker task	Response inhibition<br>Sustained Attention<br>Self- regulation of Affect
P3d	P3 difference wave<br>(No GoP3 minus GoP3)	Related to action inhibition	Go/ no-go task<br>Continue Performance Task	Response inhibition
GoP3 （No-go P3）	P3 after the Go-stimulus （No-go stimulus）	Related to action inhibition	Go/ no-go task	Response Inhibition
CueP3	P3 after the Cue stimulus	Represents attentional orienting	Go/ no-go task<br>Continue Performance Task	Response Inhibition<br>Sustained Attention

P3a	Reflect frontal lobe function	Related to initial attention neural sources allocation	Go/ no-go task	Response Inhibition
P3b	Reflect parietal lobe function	Reflects the match between the stimulus and an internal representation of a target	Go/ no-go task<br>Working Memory task	Working Memory
N2	Negative deflection occurs around 200ms after stimulus onset and has a fronto-central distribution	Accepted as reflecting conflict monitoring and detection	Flanker task<br>Go/no-go task	Sustained Attention<br>Response Inhibition
N2pc	Negative potential and emerges over the posterior scalp around 200ms after the appearance of stimuli.	An electrophysiological marker of visual attentional selection, and reflect attention deficits	Working Memory task<br>Self- regulation of Affect	Working Memory<br>Self-regulation of Affect
N2d	N2 difference wave<br>(NoGoN2 minus GoN2)	Associated with conflict detection	Go/ no-go task<br>Continue Performance Task	Response Inhibition
Error Positivity (Pe)	Positive deflection occurs about 300–500ms after an error and has a centro-parietal distribution	Linked to error awareness and response evaluation	Flanker task<br>Go/ no-go task<br>Self- regulation of Affect	Response Inhibition<br>Self-regulation of Affect
Error-related Negativity (ERN)	Negative deflection occurs about 50–100ms after incorrect responses and has a fronto-central scalp distribution	Early error detection	Flanker task<br>Go/ no-go task	Response Inhibition<br>Self-regulation of Affect
CNV	Negativity approximately 100ms before the second stimulus	Resource allocation for an upcoming target, and represents preparatory processes, including anticipatory attention	Go/ no-go task<br>Continue Performance Task	Sustained Attention<br>Response Inhibition
CDA	Emerges over the posterior scalp 400–800ms after the memory array,	Assess visual working memory capacity and may be a measure of how much information is currently in mind	Working Memory task	Working Memory

Supplement table 5. Paradigm and ERPs of Executive Function

Executive Function	Paradigm	Related ERPs
Response Inhibition	Go/ no-go task, Flanker task, Stroop task, Stop-signal task	Pe, ERN, P3, N2, CNV, Pe, P1
Sustained Attention	Oddball task, Continuous performance task, Sustained attention task, Go/no-go task	P3, N3, N2, P2, P350, N140, P1, CNV
Working Memory	Working memory task,， n-back task， Visual short memory test	CDA, N2, P3
Self-regulation of Affect	Go/no-go task, oddball task， dual valence task， verbal dot-probe task	LPP, ERN, Pe, N170, P250, P100, P1, P3, N2

Supplement table 6. PRISMA 2020 Statement and Checklist

adSection and Topic	Item #	Checklist item	Location where item is reported
**TITLE**			
Title	1	Identify the report as a systematic review.	Title
**ABSTRACT**			
Abstract	2	See the PRISMA 2020 for Abstracts checklist.	Abstract checklist
**INTRODUCTION**			
Rationale	3	Describe the rationale for the review in the context of existing knowledge.	1.Introduction
Objectives	4	Provide an explicit statement of the objective(s) or question(s) the review addresses.	1.Introduction
**METHODS**			
Eligibility criteria	5	Specify the inclusion and exclusion criteria for the review and how studies were grouped for the syntheses.	2.2 Inclusion and exclusion criteria
Information sources	6	Specify all databases, registers, websites, organisations, reference lists and other sources searched or consulted to identify studies. Specify the date when each source was last searched or consulted.	2.1 Search strategy and information sources
Search strategy	7	Present the full search strategies for all databases, registers and websites, including any filters and limits used.	2.1 Search strategy and information sources
Selection process	8	Specify the methods used to decide whether a study met the inclusion criteria of the review, including how many reviewers screened each record and each report retrieved, whether they worked independently, and if applicable, details of automation tools used in the process.	3.1 Literature search and assessment of risk of bias &Figure1
Data collection process	9	Specify the methods used to collect data from reports, including how many reviewers collected data from each report, whether they worked independently, any processes for obtaining or confirming data from study investigators, and if applicable, details of automation tools used in the process.	2.3 Data extraction
Data items	10a	List and define all outcomes for which data were sought. Specify whether all results that were compatible with each outcome domain in each study were sought (e.g. for all measures, time points, analyses), and if not, the	2.3 Data extraction

adSection and Topic	Item #	Checklist item	Location where item is reported
		methods used to decide which results to collect.	
	10b	List and define all other variables for which data were sought (e.g. participant and intervention characteristics, funding sources). Describe any assumptions made about any missing or unclear information.	2.3 Data extraction
Study risk of bias assessment	11	Specify the methods used to assess risk of bias in the included studies, including details of the tool(s) used, how many reviewers assessed each study and whether they worked independently, and if applicable, details of automation tools used in the process.	2.4 Study quality assessment
Effect measures	12	Specify for each outcome the effect measure(s) (e.g. risk ratio, mean difference) used in the synthesis or presentation of results.	3.2 General character of the study &Table 2-7 Main finding
Synthesis methods	13a	Describe the processes used to decide which studies were eligible for each synthesis (e.g. tabulating the study intervention characteristics and comparing against the planned groups for each synthesis (item #5)).	3.2 General character of the study &Table 2-7
	13b	Describe any methods required to prepare the data for presentation or synthesis, such as handling of missing summary statistics, or data conversions.	Table 2-7
	13c	Describe any methods used to tabulate or visually display results of individual studies and syntheses.	3.2.1 Resting state spectral power<br>3.2.2 Main Experimental tasks and ERPs of EFs
	13d	Describe any methods used to synthesize results and provide a rationale for the choice(s). If meta-analysis was performed, describe the model(s), method(s) to identify the presence and extent of statistical heterogeneity, and software package(s) used.	Table 2-7<br>For qualitative synthesis
	13e	Describe any methods used to explore possible causes of heterogeneity among study results (e.g. subgroup analysis, meta-regression).	Table 2-7<br>For qualitative synthesis
	13f	Describe any sensitivity analyses conducted to assess robustness of the synthesized results.	None

adSection and Topic	Item #	Checklist item	Location where item is reported
Reporting bias assessment	14	Describe any methods used to assess risk of bias due to missing results in a synthesis (arising from reporting biases).	None
Certainty assessment	15	Describe any methods used to assess certainty (or confidence) in the body of evidence for an outcome.	None
**RESULTS**			
Study selection	16a	Describe the results of the search and selection process, from the number of records identified in the search to the number of studies included in the review, ideally using a flow diagram.	3.1 Literature search and assessment of risk of bias &Figure1
	16b	Cite studies that might appear to meet the inclusion criteria, but which were excluded, and explain why they were excluded.	3.1 Literature search and assessment of risk of bias &Figure1
Study characteristics	17	Cite each included study and present its characteristics.	Table 2-7
Risk of bias in studies	18	Present assessments of risk of bias for each included study.	2.4 Study quality assessment &Supplement table 8
Results of individual studies	19	For all outcomes, present, for each study: (a) summary statistics for each group (where appropriate) and (b) an effect estimate and its precision (e.g. confidence/credible interval), ideally using structured tables or plots.	Table 2-7
Results of syntheses	20a	For each synthesis, briefly summarise the characteristics and risk of bias among contributing studies.	4. Discussion& Figure 3-6
	20b	Present results of all statistical syntheses conducted. If meta-analysis was done, present for each the summary estimate and its precision (e.g. confidence/credible interval) and measures of statistical heterogeneity. If comparing groups, describe the direction of the effect.	4. Discussion
	20c	Present results of all investigations of possible causes of heterogeneity among study results.	4. Discussion
	20d	Present results of all sensitivity analyses conducted to assess the robustness of the synthesized results.	None

adSection and Topic	Item #	Checklist item	Location where item is reported
Reporting biases	21	Present assessments of risk of bias due to missing results (arising from reporting biases) for each synthesis assessed.	None
Certainty of evidence	22	Present assessments of certainty (or confidence) in the body of evidence for each outcome assessed.	None
**DISCUSSION**			
Discussion	23a	Provide a general interpretation of the results in the context of other evidence.	4. Discussion
	23b	Discuss any limitations of the evidence included in the review.	4.5 Limitation
	23c	Discuss any limitations of the review processes used.	4.6 Conclusion and future direction
	23d	Discuss implications of the results for practice, policy, and future research.	4.6 Conclusion and future direction
**OTHER INFORMATION**			
Registration and protocol	24a	Provide registration information for the review, including register name and registration number, or state that the review was not registered.	None
	24b	Indicate where the review protocol can be accessed, or state that a protocol was not prepared.	None
	24c	Describe and explain any amendments to information provided at registration or in the protocol.	None
Support	25	Describe sources of financial or non-financial support for the review, and the role of the funders or sponsors in the review.	Funding
Competing interests	26	Declare any competing interests of review authors.	Declarations
Availability of data, code and	27	Report which of the following are publicly available and where they can be found: template data collection forms; data extracted from included studies; data used for all analyses; analytic code; any other materials used	2.1 search strategy and information sources Publicly available datasets Pubmed and Web

adSection and Topic	Item #	Checklist item	Location where item is reported
other materials		in the review.	of Science

Supplement table 7. PRISMA 2020 Abstract checklist

Section and Topic	Item #	Checklist item	Reported (Yes/No)
**TITLE**			
Title	1	Identify the report as a systematic review.	Yes
**BACKGROUND**			
Objectives	2	Provide an explicit statement of the main objective(s) or question(s) the review addresses.	Yes
**METHODS**			
Eligibility criteria	3	Specify the inclusion and exclusion criteria for the review.	Yes
Information sources	4	Specify the information sources (e.g. databases, registers) used to identify studies and the date when each was last searched.	Yes
Risk of bias	5	Specify the methods used to assess risk of bias in the included studies.	Yes
Synthesis of results	6	Specify the methods used to present and synthesise results.	Yes

Section and Topic	Item #	Checklist item	Reported (Yes/No)
**RESULTS**			
Included studies	7	Give the total number of included studies and participants and summarise relevant characteristics of studies.	Yes
Synthesis of results	8	Present results for main outcomes, preferably indicating the number of included studies and participants for each. If meta-analysis was done, report the summary estimate and confidence/credible interval. If comparing groups, indicate the direction of the effect (i.e. which group is favoured).	Yes
**DISCUSSION**			
Limitations of evidence	9	Provide a brief summary of the limitations of the evidence included in the review (e.g. study risk of bias, inconsistency and imprecision).	Yes
Interpretation	10	Provide a general interpretation of the results and important implications.	Yes
**OTHER**			
Funding	11	Specify the primary source of funding for the review.	Yes
Registration	12	Provide the register name and registration number.	No

From: Page MJ, McKenzie JE, Bossuyt PM, Boutron I, Hoffmann TC, Mulrow CD, et al. The PRISMA 2020 statement: an updated guideline for reporting systematic reviews. BMJ 2021;372:n71. doi: 10.1136/bmj.n71. This work is licensed under CC BY 4.0. To view a copy of this license, visit https://creativecommons.org/licenses/by/4.0/

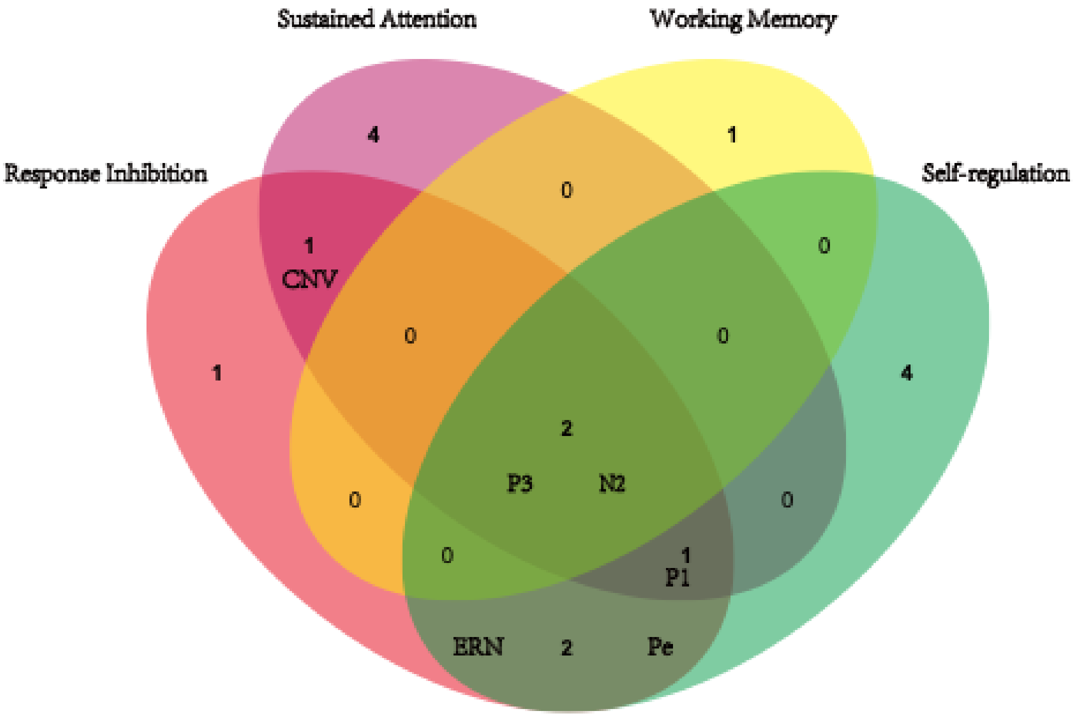

Supplement Figure 1. ERPs of Executive Function assessment

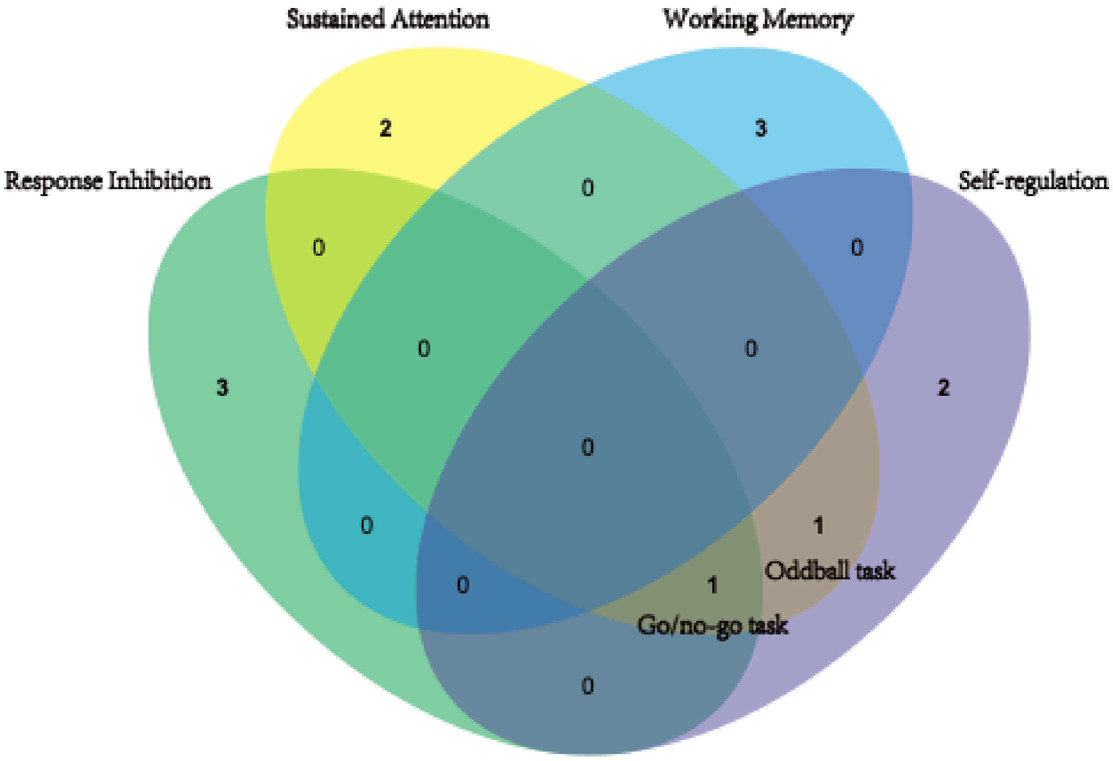

Supplement Figure 2. Experimental paradigms of Executive Function assessment

Supplement table 8. Quality assessment of included studies

Study	Selection (Maximum 4 stars)	Comparability (Maximum 2 stars)	Exposure (Maximum 3 stars)
(Kiiski, Bennett, et al. 2020)	****	*	***
(Dupuy et al. 2021a)	***	*	***
(Clarke et al. 2019)	***	*	***
(Han et al. 2022)	***		***
(Tombor, Kakuszi, et al. 2019)	**	*	***
(Li et al. 2019a)	***	*	***
(Schneidt, Hoehnle, and Schoenenberg 2020)	****	*	***
(Bresnahan and Barry 2002)	***	*	***
(Koehler et al. 2009)	***	**	***
(Liechti et al. 2013)	****	*	***
(Markovska-Simoska and Pop-Jordanova 2017)	**	*	***
(Poil et al. 2014)	***	*	***
(Woltering et al. 2012)	**	*	**
(Buyck and Wiersema 2014)	**	*	***
(Clarke et al. 2008)	***	*	***
(Kitsune et al. 2015)	**	*	***
(Skirrow et al. 2015)	**	*	***
(Yoon et al. 2024)	**	*	**
(Loo et al. 2009)	**	*	***
(Cowley, Juurmaa, and Palomäki 2022)	**		***
(Herrmann et al. 2010)	**	*	**
(Marquardt et al. 2018)	**	*	***
(Dubreuil-Vall, Ruffini, and Camprodon 2020)	**		***
(McLoughlin et al. 2009)	***	*	***
(Ehlis, Deppermann, and Fallgatter 2018)	***		*
(Papp et al. 2020)	***	*	**
(Kropotov et al. 2019)	***		***
(Rodriguez and Baylis 2007)	***	**	**
(Bozhilova et al. 2022)	**		***
(Munger et al. 2022)	**		**
(Wiersema et al. 2006)	**	*	***
(Woltering et al. 2013)	***	*	**
(Münger et al. 2021)	**		***
(Mayer, Wyckoff, and Strehl 2016)	**		***
(Wiersema, van der Meere, and Roeyers 2009)	****	*	**
(Balogh et al. 2017)	**		***
(Czobor et al. 2017)	***	*	**
(Köchel, Leutgeb, and Schienle 2012)	**		**
(O'Connell et al. 2009)	**		***
(Karch et al. 2012)	**	*	**

(Smit et al. 2023a)	**		**
(Luo et al. 2019)	***	*	**
(Jang, Kim, and Kim 2020)	**	*	**
(Ibáñez et al. 2011)	**	*	***
(Thoma et al. 2020)	**	*	**
(Herrmann et al. 2009)	***	*	***
(Shushakova, Ohrmann, and Pedersen 2018)	****	**	**
(Salomone et al. 2020)	**		***
(Marzinzik et al. 2012)	**	*	***
(Raz and Dan 2015a)	**	*	**
(Barry et al. 2009)	***	*	**
(Itagaki et al. 2011)	*		**
(Leroy et al. 2018)	***		***
(Kaur et al. 2019)	**	*	***
(Dhar et al. 2010)	**	*	**
(Freichel et al. 2024b)	**		**
(Doehnert et al. 2013)	**		***
(Cheung et al. 2017)	**		***
(Mauriello et al. 2022)	***	*	***
(Gumenyuk et al. 2023)	**	*	***
(Schneidt et al. 2018)	**	*	**
(Hasler et al. 2016)	**		***
(Wiegand et al. 2016)	****	**	***
(Spronk, Vogel, and Jonkman 2013)	****	**	**

"*" Represents one score

Supplement table 9. Criteria for modified Newcastle Ottawa Scale

Bias domain	Signaling question	Response options
Selection	1.Is the case definition adequate	1.Yes, standardized diagnostic tools used (*)<br>2.Yes, reliance on self-report scales or unclear diagnostic methods<br>3. No description
	2.Representativeness of the cases	1.Consecutive or obviously representative series of cases (*)<br>2.Potential for selection biases or not stated
	3.Selection of Controls	1. Control recruited from the same population as ADHD group (*)<br>2.Control source unclear or significantly different
	4.Definition of Controls	1. Controls excluded for psychiatric disorders via structured interviews (*)<br>2. Only self-reported "no psychiatric history."
Comparability	5.Conparability of cases and controls based on the most important factors	1. Study controls for age, sex (*)
	6. Comparability of cases and controls based on the second important factors	1.Study controls for ADHD subtype (*)

Exposure	7. EEG data acquisition &preprocessing	1. Explicit sampling rate, bandpass filtering, and artifact removal (*)<br>2. Incomplete parameters or vague methods
	8. Standardization of EEG data collection condition	1. Controlled EEG recording conditions, lab environment or experimental paradigm (*)<br>2. Not mention or large variability
	9. Medication usage control	1. Specify ADHD medication usage and time of Interruption (*)<br>2. Failure to provide detailed medication information or discontinuation period
